# Changes in joint, muscle, and tendon stiffness following repeated hopping exercise

**DOI:** 10.14814/phy2.14237

**Published:** 2019-10-12

**Authors:** Keitaro Kubo, Toshihiro Ikebukuro

**Affiliations:** ^1^ Department of Life Science The University of Tokyo Meguro Tokyo Japan

**Keywords:** Plantar flexor muscles, drop jump, range of motion, ultrasonography

## Abstract

The purpose of this study was to elucidate the mechanisms of decline in joint stiffness after repeated stretch‐shortening cycle exercises according to changes in both muscle‐tendon properties and neuromuscular activities. Eleven males performed fatigue task (5 sets of 50 hopping). Ankle joint stiffness and electromyographic activities (mEMG) of plantar flexor and tibial anterior muscles during drop jump were measured before and after fatigue task. Active muscle stiffness with (100 deg·sec^−1^) and without (250 deg·sec^−1^) stretch reflex were calculated according to changes in estimated muscle force and fascicle length during fast stretching after submaximal isometric contractions. Tendon stiffness was measured during ramp and ballistic contractions. After fatigue task, joint stiffness significantly decreased by 20.7 %, whereas mEMG of measured muscles during drop jump did not. After fatigue task, active muscle stiffness with and without stretch reflex significantly decreased by 15.7 % and 21.5 %, and tendon stiffness measured during ramp and ballistic contractions did not change. In addition, the relative change in joint stiffness was significantly correlated with that in active muscle stiffness with stretch reflex (*r* = 0.737, *P* = 0.009), but not with those in the other measured variables. These results suggested that the decline in joint stiffness after repeated hopping exercises would be caused by changes in active muscle stiffness, but not those in tendon properties or neuromuscular activities.

## Introduction

It has been shown that repeated stretch‐shortening cycle (SSC) exercises lead to an acute reduction in joint stiffness (change in joint torque divided by change in joint angle) (Horira et al., 1996; Horita et al., 1999; Kuitunen et al., 2002; Toumi et al., 2006; Lazaridis et al., 2018). In these studies, however, changes in the mechanical properties of muscles and tendons could not be assessed separately. In the last two dacades, several studies have investigated acute and chronic changes in stiffness and hysteresis of human tendons following various kinds of exercises (*e.g.,* Kubo et al., 2017). To date, the mechanical properties of tendons have been shown to remain unchanged after repeated SSC exercises, for example, hopping and endurance running (Kubo et al., 2005; Peltonen et al., 2010; Farris et al., 2012; Peltonen et al., 2012). In these studies, the elongation rates of tendons during the measurements of tendon properties were markedly lower than those during SSC exercises. Our recent studies showed that tendon properties (elongation and hysteresis) during ballistic contractions were different from those during ramp contractions (Kubo et al., 2017; Kubo, 2018; Kouno et al., 2019). Accordingly, we need to revalidate the effects of repeated SSC exercises on tendon properties during ballistic as well as ramp contractions.

In addition to the changes in tendon properties mentioned above, the mechanical properties of muscles need to be determined in order to clarify acute changes in joint stiffness after repeated SSC exercises. To date, the mechanical properties of human muscle *in vivo* have been investigated under passive conditions during slow stretching (Morse et al., 2008; Nakamura et al., 2012). We demonstrated that muscle stiffness during submaximal isometric contractions (*i.e.,* active muscle stiffness) could be evaluated according to changes in the torque and fascicle length during fast stretching (peak angular velocity was 250 deg·sec^−1^) (Kubo, 2014). In order to avoid any potential stretch reflex, we analyzed points when the ankle joint commenced movement and then 60 msec thereafter (Blanpied and Smidt, 1992; Kubo, 2014). Therefore, the active muscle stiffness obtained using this methodology represented the intrinsic characteristic of muscle under an active condition without any potential neural effects. According to cross‐sectional and longitudinal studies (Kubo et al., 2015; Kubo et al., 2017), we have investigated training‐induced changes in active muscle stiffness. Furthermore, we recently proposed a method to evaluate active muscle stiffness including the effect of the stretch reflex from changes in the torque and fascicle length during slower stretch velocity (peak angular velocity was 100 deg·sec^−1^) (Kubo et al., 2018). Previous studies showed that the relative change in joint stiffness after exhausting SSC exercises was significantly correlated with that in the electromechanical activities and stretch reflex during the drop jump (Horira et al., 1996; Avela et al., 1999; Kuitunen et al., 2002). Therefore, it is likely that after repeated SSC exercises, active muscle stiffness with the stretch reflex decreases more conspicuously compared with that without the stretch reflex.

Since 2000, ultrasonography has been used to clarify the significant role of the muscle‐tendon interaction during SSC exersises throughout investigating the relationship between the muscle‐tendon properties and the SSC performence and the behavior of muscle fibers (fascicles) and tendons (*e.g.,* Kawakami et al., 2002; Sousa et al., 2007). Furthermore, recent studies showed the effects of training‐induced changes in tendon stiffness on muscle‐tendon behavior during landing and depth jumping (Hirayama et al., 2017; Werkhausen et al., 2018). The findings on changes in muscle‐tendon behavior during SSC exercises might support further this notion, which the performances during SSC exercises were influenced by the muscle‐tendon properties. Therefore, it is necessary to determine changes in muscle‐tendon behavior during the measurement of joint stiffness (*i.e.,* drop jump) in order to directly clarify the mechanisms of a decline in joint stiffness after repeated SSC exercises.

In the previous studies as mentioned earlier, the contribution of each joint during the measurement of joint stiffness (*i.e.,* drop jump) would change after repeated SSC exercises, since these jump exercises consist of multi‐joint movements. Indeed, Kuitunen et al. (2002) considered that subjects would minimize the fatigue‐induced reduction in jump performance by increasing the contribution of hip extensor muscles, since an increase in hip angular displacement after a fatigue task occurred during jumping. Therefore, we must adopt jump tests using a single joint in order to minimize the contribution of other joints and muscles if fatigue‐induced changes in joint stiffness are to be correctly evaluated. In this study, we determined changes in the mechanical properties of muscle‐tendon complex (joint, passive muscle, active muscle, and tendon stiffness), neuromuscular activity and behavior of fascicles during the drop jump (*i.e.,* measurement of joint stiffness) after repeated SSC exercises using a single joint. The purpose of this study was to elucidate the mechanisms of the decline in joint stiffness after repeated SSC exercises according to changes in both muscle‐tendon properties and neuromuscular activities. We hypothesized that a fatigue‐induced decline in joint stiffness would be associated with decreases in active muscle stiffness with the stretch reflex and muscle activities (including the stretch reflex) during jumping.

## Methods

### Subjects

The sample size was estimated using the data from previous studies (Horira et al., 1996; Lazaridis et al., 2018) in which the effect of repeated SSC exercises on joint stiffness were determined. On the basis of an *α* level of 0.05 and a power (1 – *β*) of 0.8, it was shown that as least nine subjects were necessary for this study. Eleven healthy males (age: 29.2 ± 10.5 years, height: 171.9 ± 5.1 cm, body mass: 69.5 ± 11.7 kg, mean ± SD) voluntarily participated in the present study. They were asked to avoid intense exercises for 3 days before the experiments. They were fully informed of the procedures to be utilized as well as the purpose of this study. Written informed consent was obtained from all subjects. This study was approved by the office of the Department of Sports Sciences, The University of Tokyo, and complied with their requirements for human experimentation.

### Experimental design

Subjects were initially familiarized with the testing procedures (see below) 1 week before the experiments. They performed two tests on 2 separate days, with at least 1 week between sessions and no longer than 2 weeks separating the two sessions. The order of the two experimental conditions (once under experimental condition and once under control condition) was randomized for each subject. The neuromuscular activity and behavior of fascicles during the drop jump (*i.e.,* measurement of joint stiffness) and the mechanical properties of muscle‐tendon complex were measured before and after the fatigue task (see below).

### Fatigue task

Subjects performed repeated unilateral hopping exercises using only the ankle joint on a sledge apparatus (VR‐4100, Cybex Corp., USA). The load used was 50% of the body mass for each subject. They initially maintained a maximal plantar flexed position. They then exerted plantar flexion torque to maximal dorsiflexion, and rebounded to start plantar flexion until the toe finally lifted away from the footplate of this apparatus. These movements were repeated without a break. The fatigue task consisted of 5 sets of 50 hopping exercises with a rest interval of 1 min. The subjects were requested to jump as high as possible.

### Joint stiffness and fascicle length during drop jump

Subjects performed a unilateral drop jump using only the ankle joint on a sledge apparatus, as described in our previous studies (Kubo et al., 2007; Kubo et al., 2017; Suzuki et al., 2019). The load used was 50% of the body mass for each subject. The vertical component of the ground reaction force (Fz) was recorded from a force plate (Kistler, 9281B, Switzerland) attached to the force platform of the apparatus. Retroreflective marks were placed on the fifth metatarsophalangeal joint, lateral malleolus, the center of rotation of the knee, and trochanter major. During jumping, subjects were filmed with a digital high‐speed video camera at a sampling frequency of 250 Hz (VCC‐H1600C, Digimo, Tokyo, Japan).

Subjects had adequate practice (submaximal jumps) to become accustomed to the test procedure before testing. The test was repeated five times before the fatigue task and three times after the fatigue task per subject, with at least 1 min between trials. The sliding table of this apparatus was moved to a height of 20 cm from the surface of the force plate to the sole of their foot with the assistance of an experimenter. Subjects were dropped from a height of 20 cm. After landing on the edge of the force plate, they arrested downward motion by eccentric plantar flexion. They then started concentric plantar flexion and took off. We excluded trials with slight knee joint flexion (>5 deg) based on images taken by the high‐speed video camera. The measured values were the means of two trials with the highest and second highest jump heights according to the flight time.

The ankle joint angle was measured using open‐source image analysis software (ImageJ, NIH, Bethesda, MD, USA). The range of motion of the ankle from touchdown to the lowest position (ROM) was calculated. The ankle joint torque during the drop jump was estimated from the following equation (Kawakami et al., 2002; Kubo et al., 2007; Kubo et al., 2017; Suzuki et al., 2019):

$$\text{Ankle joint torque}=\text{Fz}\cdot \text{L1}\cdot \cos(A_{J})$$

where Fz, L1, and A_J_ are the vertical component of the ground reaction force, the length from the estimated center of ankle joint to the ball of the foot, and the ankle joint angle, respectively. Ankle joint stiffness was calculated as the change in joint torque divided by the change in the ankle joint angle during the eccentric phase (Kubo et al., 2007; Kubo et al., 2017; Suzuki et al., 2019). The repeatability of measurement of joint stiffness was investigated on 2 separate days (measured before experimental and control conditions). The intraclass correlation coefficient and coefficient of variation were 10.5% and 0.874, respectively.

A real‐time ultrasonic apparatus (Prosound α7, Hitachi Aloka Medical, Tokyo, Japan) was used to continuously record longitudinal ultrasonic images of the medial gastrocnemius muscle (MG) during the exercises. At 30% of the lower leg length (i.e., distance from the center of the malleolus lateralis to the articular cleft between the femur and tibiacondyles), the scanning probe (7.5‐MHz wave frequency with an 80‐mm scanning length; UST 5712, Aloka, Tokyo, Japan) of the apparatus was secured with adhesive tape on the skin. Ultrasonic images were recorded on a video tape at 60 Hz, synchronized with recordings of a clock timer for subsequent analysis. The fascicle length was defined as the distance between the insertions of the fascicle into the superficial and deep aponeurosis. We analyzed the fascicle length of MG from pre‐landing (defined as 100 msec preceding landing) to when the toe lifted away from the force plate.

In addition, we recorded electromyographic activities (EMG) from the lateral gastrocnemius muscle (LG), soleus muscle (SOL), and tibial anterior muscle (TA) with bipolar surface electrodes (5 mm in diameter) placed on the belly of each muscle with a 25‐mm interelectrode distance. The electrodes were connected to a preamplifier and differential amplifier with a bandwidth of 5 to 500 Hz (MEG‐6116; Nihon Koden, Tokyo, Japan). EMG signals were transmitted to a computer at a sampling rate of 1 kHz. EMG was full‐wave rectified and averaged for the duration of the contraction (mEMG). During jumping tests, mEMG values from LG, SOL, and TA were calculated from the pre‐landing, eccentric, and concentric phases, respectively, according to the ankle joint angle. In addition, the mean mEMG in LG and SOL was defined as the mEMG of the plantar flexor muscles (PF).

### Passive muscle stiffness

The joint angle, passive torque, and fascicle length were measured to assess passive muscle stiffness (Kubo, 2014; Kubo et al., 2015; Kubo et al., 2017). Subjects lay prone on a test bench with a foot tightly secured by two straps to the footplate of a specially designed dynamometer (AO‐K01, Applied Office, Tokyo, Japan). The ankle joint was set at 100 deg (with the foot perpendicular to the tibia = 90 deg with angles more than 90 deg in plantar flexion) with the knee joint at full extension. While subjects maintained completely relaxed muscles, the ankle was passively moved from 100 to 80 deg at a constant velocity of 5 deg·sec^‐1^. In order to minimize thixotropic effects as preconditioning (Muraoka et al., 2002; Hoang et al., 2007), we collected data during the 6th cycle after 5 cycles. Passive torque (TQ) measured during slow stretching was converted to muscle force (Fm) using the following equation:

$$F_{m}=k\cdot \text{TQ}\cdot {\text{MA}}^{-1}$$

where *k* represents the relative contribution of the physiological cross‐sectional area of MG within PF (16%; Fukunaga et al., 1996), and MA is the moment arm length of the triceps surae muscles at 90 deg of the ankle joint (50 mm; Maganaris et al., 1998).

A real‐time ultrasonic apparatus (SSD‐6500, Aloka, Tokyo, Japan) was used to obtain a longitudinal ultrasonic image of MG at the level of 30% of the lower leg length during slow stretching. Ultrasonic images were recorded on a video tape at 30 Hz and synchronized with recordings of a clock timer for subsequent analyses. The fascicle length was defined as the distance between the insertion of the fascicle into the superficial and deep aponeuroses.

The passive torque, joint angle, and fascicle length were continuously recorded during slow stretching. The slope of the portion of passive muscle force—fascicle length curve from 90 to 80 deg, was defined as passive muscle stiffness (Kubo et al., 2015; Kubo et al., 2017). The repeatability of measurement of passive muscle stiffness was investigated on 2 separate days (measured before experimental and control conditions). The intraclass correlation coefficient and coefficient of variation were 10.5% and 0.897, respectively.

### Active muscle stiffness

The posture of the subject and setup were similar to those for the measurement of passive muscle stiffness, as described above. After a standardized warm‐up, subjects performed two or three maximal voluntary isometric contractions (MVC) at a 100‐deg ankle angle with a 2‐min rest period between each trial. The peak torque value within all trials was recorded as the MVC value.

After a 5‐min rest period, measurements of active muscle stiffness were performed using a previously described procedure (Kubo et al., 2018). A specially designed dynamometer was programmed to apply dorsiflexion stretches from 100 to 80 deg. In this study, the measurements of active muscle stiffness were carried out at two angular velocities (peak angular velocities were 250 and 100 deg·sec^−1^). For the 250‐deg·sec^−1^ condition, a 60‐ms period after stretch onset was analyzed in order to avoid any neural effects (Blanpied and Smidt, 1992; Kubo, 2014). During this period (60ms), the range of motion was about 8 deg, and the angular velocity reached about 250 deg·sec^−1^. For the 100‐deg·sec^−1^ condition, a 110‐msec period after stretch onset was analyzed in order to include any neural effects (short‐ and long‐latency stretch reflexes). During this period (110 msec), the range of motion was about 8 deg, and the angular velocity reached about 100 deg·sec^−1^.

An additional measurement was performed two times at 0% MVC before the short‐range stretch experiment for data‐correction purposes. The averaged torque during the relaxed condition (caused by inertia and passive elasticity) was subtracted from the measured torque during each of the active stretch trials (e.g., Kubo, 2014). The measurement of active muscle stiffness was performed two times per condition (250 and 100 deg·sec^−1^) at 50% of MVC with the visual aid of exerted torque on an oscilloscope. The measured values were the means of two trials.

During the measurement of active muscle stiffness, the fascicle length of MG was measured using a real‐time ultrasonic apparatus (SSD‐6500, Aloka, Tokyo, Japan). At the level of 30% of the lower leg length, the scanning probe of the apparatus was fastened using adhesive tape on the skin. Ultrasonic images were stored at 98 Hz in the computer memory of the apparatus (e.g., Kubo, 2014). An electric signal was superimposed on the images to synchronize them with the torque, joint angle, and electromyographic activity (see below). The slope of muscle force—fascicle length within the period analyzed, as described earlier, was defined as the active muscle stiffness (Kubo, 2014; Kubo et al., 2015; Kubo et al., 2017; Kubo et al., 2018). The repeatability of measurement of active muscle stiffness was investigated on 2 separate days (measured before experimental and control conditions). The intraclass correlation coefficient and coefficient of variation were 11.8% and 0.893 under the 250‐deg·sec^−1^ condition and 9.2% and 0.853 under the 100‐deg·sec^−1^ condition, respectively.

### Stiffness of tendon structures

Each subject lay prone on the test bench of a dynamometer (custom made, VINE, Tokyo, Japan), with the right foot tightly secured to the footplate of the dynamometer by two straps. The ankle joint was set in a neutral anatomical position (90 deg) with the knee joint at full extension. After a warm‐up and submaximal contractions to become accustomed to the tests, subjects were asked to exert isometric plantar flexion torque at two different speeds of contraction, that is, ramp and ballistic contractions. For ramp contractions, they generated increasing torque from zero (relaxation) to MVC within 5 sec (e.g., Kubo et al., 2015). For ballistic contractions, they exerted torque as powerfully and quickly as possible (Kubo et al., 2017; Kubo, 2018; Kouno et al., 2019). The measurement was repeated two times for each task (ranp and ballistic contractions) with at least one minute between trials. Torque data were collected at a sampling rate of 1 kHz for further analysis.

A longitudinal ultrasonic image of MG during contractions was obtained using real‐time ultrasonic apparatus (SSD‐6500, Aloka, Tokyo, Japan). Ultrasonic images were recorded as video signals at 60 Hz with a videotape recorder and synchronized with the torque and joint angle data by superimposing a clock timer for subsequent analyses. To assess the elongation of tendon structures (including outer tendon and aponeurosis), the movement of the intersection made by one fascicle and aponeurosis was measured. Even during isometric contraction, angular joint rotation occurred in the direction of ankle plantar flexion, resulting in the tendon displacement (Magnusson et al., 2001). To measure ankle joint angle during isometric contraction, an electrical goniometer (Penny and Giles, Newport, UK) was placed on the lateral aspect of the ankle. Additional measurements were executed under passive conditions in order to correct the measurements taken for the elongation of tendon structures. The movement of the intersection made by one fascicle and aponeurosis for the influence of rotating the ankle from 90 to 99 deg was measured, and was subtracted from the measured elongation of tendon structures during isometric contraction (Magnusson et al., 2001; Kubo et al., 2017).

The slope of the relationship between muscle force and the elongation of tendon structures from 50 to 100% of MVC was defined as the stiffness of tendon structures (e.g., Kubo et al., 2017). The repeatability of measurement of tendon stiffness was investigated on 2 separate days (measured before experimental and control conditions). The intraclass correlation coefficient and coefficient of variation were 8.2% and 0.924 for ramp contraction and 11.1% and 0.909 for ballistic contraction, respectively.

### Statistical analysis

Descriptive data are reported as means ± standard deviation. A two‐way ANOVA with repeated measures {2 (conditions) x 2 (test times)} and a post hoc analysis were used to analyze data. The F ratios for main effects and interactions were considered to be significant. Significant differences among means were detected using a Bonferroni post hoc test. In ANOVA, Mauchly's sphericity test was performed to assess the homogeneity of variance. Greenhouse‐Geisser correction was applied where assumption of sphericity was violated. Pearson's product‐moment correlation was computed to assess the relationships among the measured variables. The level of significance was set at *P* < 0.05 for all tests. Statistical computations were performed using IBM SPSS Statistics (version 19).

## Results

Table 1 shows the measured variables during the measurement of joint stiffness (i.e., drop jump) before and after the fatigue task. Although the duration of contact significantly increased after the fatigue task, the angular velocities during eccentric and concentric phases did not change. For PF and TA, mEMG during pre‐landing, eccentric, and concentric phases did not change after the fatigue task. The peak ankle joint torque tended to decrease (*P* = 0.060), ROM significantly increased (*P* = 0.004), and the joint stiffness significantly decreased by 20.7% (*P* = 0.003) after the fatigue task. The relative change in joint stiffness was correlated with that in ROM (*r* = −0.892, *P* < 0.001) but not peak torque (*r* = 0.318, *P* = 0.341).

**Table 1:** Measured variables during the measurement of joint stiffness (i.e., drop jump). Mean (SD).

	Experimental condition		Control condition	
	Before	After^a^	Before	After
Duration of contact (msec)	338.7 (34.9)	385.2 (38.5) ***	342.0 (36.6)	336.0 (37.1)
Angular velocity during eccentric phase (deg·sec^−1^)	128.3 (27.6)	137.1 (28.8)	134.9 (28.8)	135.4 (27.4)
Angular velocity during concentric phase (deg·sec^−1^)	184.2 (26.6)	166.2 (35.6)	184.6 (33.0)	187.9 (26.9)
mEMG of PF during prelanding (mV·sec^−1^)	0.087 (0.045)	0.086 (0.045)	0.099 (0.057)	0.081 (0.077)
mEMG of PF during eccentric phase (mV·sec^−1^)	0.200 (0.063)	0.217 (0.060)	0.194 (0.051)	0.206 (0.072)
mEMG of PF during concentric phase (mV·sec^−1^)	0.188 (0.069)	0.184 (0.073)	0.202 (0.076)	0.198 (0.059)
mEMG of TA during prelanding (mV·sec^−1^)	0.075 (0.044)	0.052 (0.035)	0.064 (0.032)	0.074 (0.048)
mEMG of TA during eccentric phase (mV·sec^−1^)	0.098 (0.073)	0.072 (0.037)	0.113 (0.058)	0.109 (0.022)
mEMG of TA during concentric phase (mV·sec^−1^)	0.089 (0.040)	0.055 (0.013)	0.070 (0.039)	0.094 (0.046)
Peak torque during eccentric phase (Nm)	261.6 (32.3)	248.3 (33.7)	257.7 (36.4)	260.3 (32.2)
Range of motion during eccentric phase (deg)	21.3 (5.5)	24.0 (5.0) **	22.6 (6.5)	22.9 (5.9)
Joint stiffness (Nm·deg^−1^)	13.7 (4.0)	10.9 (2.9) **	12.3 (3.8)	12.1 (3.0)
Fascicle shortening during eccentric phase (mm)	5.8 (3.4)	1.0 (2.8) ***	6.3 (2.0)	6.7 (2.3)
Fascicle shortening during concentric phase (mm)	6.8 (2.6)	7.9 (3.3)	6.5 (1.6)	7.2 (2.5)

MEMG, mean electromyographic activities, PF, plantar flexor muscles, TA, tibial anterior muscle.

a Significantly different from before (**P < 0.01, ***P < 0.001).

**Figure 1:**
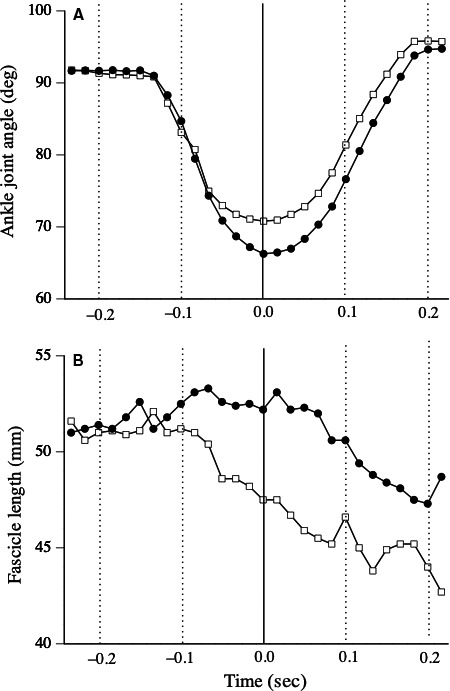
Typical example of behavior of medial gastrocnemius muscle fascicle during drop jump (*i.e.,* measurement of joint stiffness) before (dotted line) and after (slid line) fatigue task.

A typical example of changes in fascicle length during the drop jump is shown in Figure 1. Although the fascicle length shortened during the eccentric phase before and after the fatigue task, the amount of fascicle shortening significantly decreased after the fatigue task (*P* < 0.001) (Table 1). During the concentric phase, the amount of fascicle shortening did not change after the fatigue task (*P* = 0.333) (Table 1).

**Figure 2:**
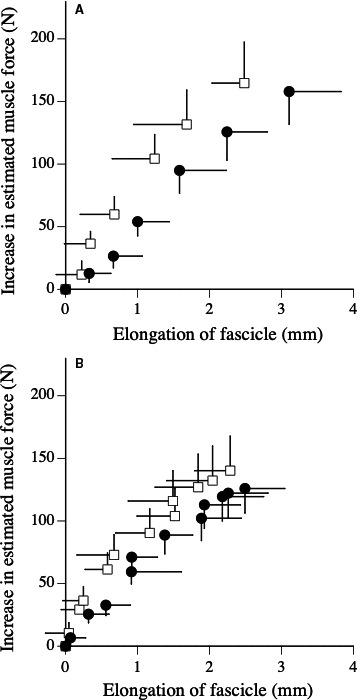
Relationships between increase in estimated muscle force and elongation of fascicle during fast stretching (A: measurement of active muscle stiffness under 250 deg·sec^−1^ condition, B: measurement of active muscle stiffness under 100 deg·sec^−1^ condition) before (open) and after (closed) fatigue task.

The relationships between an increase in the estimated muscle force and elongation of fascicle during the measurements of active muscle stiffness are shown in Figure 2. Increases in the estimated muscle force during fast stretching significantly decreased after the fatigue task under the 100‐deg·sec^−1^ condition (*P* = 0.037), but not the 250‐deg·sec^−1^ condition (*P* = 0.160) (Table 2). The increase in the fascicle length during fast stretching significantly increased after the fatigue task under the 250‐deg·sec^−1^ condition (*P* < 0.001), but not 100‐deg·sec^−1^ condition (*P* = 0.090) (Table 2). Active muscle stiffness significantly decreased by 21.5% under the 250‐deg·sec^−1^ condition (*P* = 0.001) and 15.7% under the 100‐deg·sec^−1^ condition (*P* = 0.024) (Table 2).

**Table 2:** Mechanical properties of muscle and tendon structures. Mean (SD).

	Experimental condition		Control condition	
	Before	After^a^	Before	After
Increase in estimated muscle force during 250 deg·sec^−1^ (N)	164.7 (32.7)	157.9 (26.0)	173.7 (35.8)	173.1 (30.3)
Increase in fascicle length during 250 deg·sec^−1^ (mm)	2.5 (0.4)	3.1 (0.7) ***	2.7 (1.1)	2.5 (0.7)
Active muscle stiffness during 250 deg·sec^−1^ (N·mm^−1^)	76.6 (19.4)	59.2 (13.3) **	85.2 (33.7)	84.2 (25.9)
Increase in estimated muscle force during 100 deg·sec^−1^ (*N*)	140.2 (27.5)	125.9 (19.50) *	144.0 (29.4)	139.6 (24.8)
Increase in fascicle length during 100 deg·sec^−1^ (mm)	2.3 (0.5)	2.5 (0.6)	2.4 (0.7)	2.4 (0.6)
Active muscle stiffness during 100 deg·sec^−1^ (N·mm^−1^)	71.0 (17.2)	58.3 (12.0) *	72.5 (19.8)	69.3 (12.6)
Peak passive muscle force during slow stretching (*N*)	60.5 (14.0)	69.6 (18.0) *	65.6 (15.7)	64.3 (14.5)
Maximal elongation of fascicle during slow stretching (mm)	11.0 (2.4)	9.7 (1.8) *	9.5 (2.2)	10.0 (2.2)
Passive muscle stiffness (N·mm^−1^)	10.3 (4.2)	16.3 (11.0) **	11.4 (4.2)	11.6 (5.9)
Maximal elongation of tendon structures during ramp contraction (mm)	18.5 (3.8)	17.0 (3.3)	18.6 (2.3)	17.9 (2.6)
Stiffness of tendon structures during ramp contraction (N·mm^−1^)	23.4 (4.7)	22.0 (7.3)	24.4 (8.2)	26.0 (10.1)
Maximal elongation of tendon structures during ballistic contraction (mm)	15.6 (3.7)	12.8 (2.6) ***	15.6 (3.49)	15.4 (4.4)
Stiffness of tendon structures during ballistic contraction (N·mm^−1^)	23.0 (4.9)	23.3 (4.4)	26.0 (9.6)	24.1 (6.8)

a Significantly different from before (*P < 0.05, **P < 0.01, ***P < 0.001).

**Figure 3:**
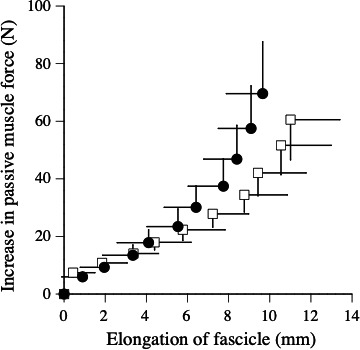
Relationships between increase in passive muscle force and elongation of fascicle during slow stretching (*i.e.,* measurement of passive muscle stiffness) before (open) and after (closed) fatigue task.

The relationship between passive muscle force and elongation of fascicle during slow stretching is shown in Figure 3. The peak passive muscle force significantly increased by 16.9% (*P* = 0.032), the elongation of fascicle significantly decreased by 11.2% (*P* = 0.011), and the passive muscle stiffness significantly increased by 32.5% (*P* = 0.003) after the fatigue task (Table 2).

**Figure 4:**
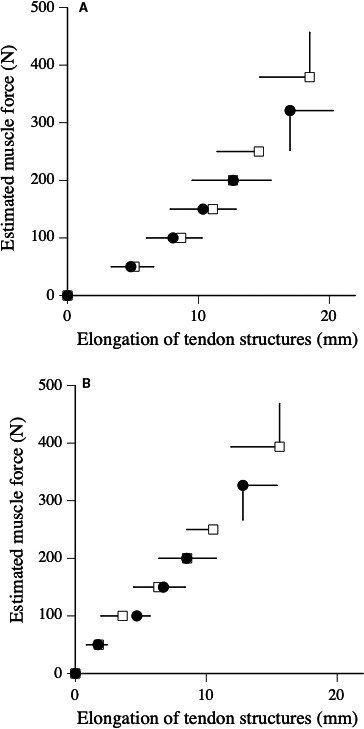
Relationships between estimated muscle force and elongation of tendon structures during ramp (A) and ballistic (B) contractions before (open) and after (closed) fatigue task.

For ramp and ballistic contractions, no significant differences in elongation of tendon structures at any force levels were found between before and after the fatigue task (Fig. 4). For ramp and ballistic contractions, the stiffness of tendon structures did not change after the fatigue task (Table 2).

**Figure 5:**
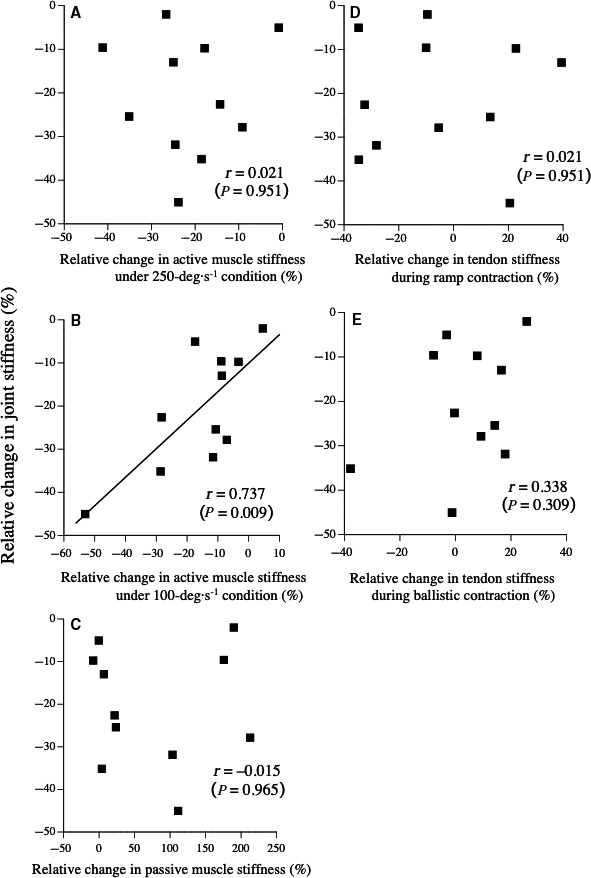
Relationships between relative change in joint stiffness and that in active muscle stiffness without stretch reflex (under 250‐deg·sec^−1^ condition) (A), active muscle stiffness with stretch reflex (under 100‐deg·sec^−1^ condition) (B), passive muscle stiffness (C), tendon stiffness during ramp contraction (D), and tendon stiffness during ballistic contraction (E)

The relative change in joint stiffness was significantly correlated with that in active muscle stiffness under the 100‐deg·sec^−1^ condition (*r* = 0.737, *P* = 0.009), but not with that in the other measured variables (Fig. 5).

Under the control condition, no significant changes were noted in the measured variables (Tables 1 and 2).

## Discussion

The main results of the present study were that active muscle stiffness measured under 250‐ and 100‐deg·sec^−1^ conditions as well as joint stiffness significantly decreased after repeated hopping exercises, whereas tendon stiffness measured during ramp and ballistic contractions and neuromuscular activities during the measurement of joint stiffness did not. Furthermore, the relative change in joint stiffness was significantly correlated with that in active muscle stiffness measured under the 100‐deg·sec^−1^ condition, but not with the other variables. Our hypotheses supported the present results, except for the fatigue‐induced decline in muscle activities during the measurement of joint stiffness.

In this study, joint stiffness significantly decreased by approximately 20% after the fatigue task. Previous studies reported significant declines (20–30%) in leg and joint stiffness after submaximal and maximal SSC exercises (Horira et al., 1996; Horita et al., 1999; Kuitunen et al., 2002; Lazaridis et al., 2018). Therefore, the fatigue task in the present study (5 sets of 50 hopping exercises) may have caused fatigue to the same extent as in the previous studies quoted earlier. In addition, the relative change in joint stiffness was correlated with that in ROM, but not peak torque. This result agrees with our recent study (Suzuki et al., 2019). Horita et al. (1999) also reported that the relative change in knee joint stiffness was negatively associated with a change in the knee joint angle after repeated drop jumps. The mechanisms of the increase in ROM during the drop jump after repeated SSC exercises are discussed in detail in the following section.

After repeated SSC exercises, the active muscle stiffness values measured under the 250‐deg·sec^−1^ condition significantly decreased, whereas the muscle activities during drop jump and tendon properties did not change (see below). Therefore, decline in active muscle stiffness would lead to an increase in ROM during the measurement of joint stiffness. The results on active muscle stiffness under the 250‐deg·sec^−1^ condition suggest that intrinsic muscle properties without the effect of the stretch reflex changed after repeated SSC exercises. Dr. Komi's colleagues demonstrated that exercise‐induced muscle damage that resulted in changes in the neural effect through modulation of the stretch reflex would be associated with the regulation of joint stiffness (Horira et al., 1996; Kuitunen et al., 2002). However, the muscle activities during the measurement of joint stiffness did not change in the present study. A possible reason for this discrepancy would be the difference in exercise mode (multi‐joint exercise in the previous studies, single‐joint exercise). Considering the present results, therefore, a decline in joint stiffness after repeated SSC exercises would be related to changes in intrinsic muscle properties (*e.g.,* the mechanical propeties of cross‐bridges and titin filaments within the sarcomere), but not to those in the neural drive and neuromuscular activities. Indeed, Trappe et al. (2002) reported that titin and nebulin content in muscle biopsies from the human vastus lateralis muscle was significantly reduced after high‐intensity eccentric knee extensor resistance exercises.

In addition, the active muscle stiffness under the 100‐deg·sec^−1^ condition (*i.e* *.,* active muscle stiffness with the stretch reflex) decreased to the same degree as for the 250‐deg·sec^−1^ condition (*i.e* *.,* active muscle stiffness without the stretch reflex) after repeated SSC exercises. Furthermore, the relative change in joint stiffness was highly correlated with that in active muscle stiffness measured under the 100‐deg·sec^−1^ condition, but not that under the 250‐deg·sec^−1^ condition (Fig. 5). Considering these findings, the change in active muscle stiffness with the stretch reflex may be closely related to the change in joint stiffness, whereas the muscle activities and stretch reflex during the measurement of joint stiffness (i.e., drop jump) did not change after repeated SSC exercises. The mean angular velocity during the eccentric phase of the drop jump (approximately 130 deg·sec^−1^) was close to that under the 100‐deg·sec^−1^ condition. Therefore, we cannot deny that the change in active muscle stiffness was influenced by the angular velocity, and not the stretch reflex. At present, we are investigating the effect of the angular velocity on active muscle stiffness in our laboratory.

The mechanical properties of tendon structures measured during ramp and ballistic contractions did not change after repeated SSC exercises. Previous studies demonstrated that the elongation and stiffness of tendons measured during ramp contractions did not change after repeated drop jumping and endurance running (Kubo et al., 2005; Peltonen et al., 2010; Peltonen et al., 2012). In the present study, we newly confirmed this point through the measurement of tendon properties by ballistic contraction. Farris et al. (2012) also reported that stiffness of the Achilles tendon determined by displacement of the muscle‐tendon junction of the medial gastrocnemius muscle during hopping did not change after 30‐min running. Considering the previous and present findings, the tendon properties did not change after repeated SSC exercises involving high‐force eccentric muscle contractions with a short duration. Accordingly, it is likely that any decline in joint stiffness following repeated SSC exercises is not related to changes in the mechanical properties of tendon strructures.

Before the fatigue task, the fascicles of MG shortened during the eccentric phase of the drop jump, whereas the whole muscle‐tendon complex lengthened (Fig. 1). Previous researchers also demonstrated that MG fascicles shortened during the eccentric phase of the drop jump and the former half of the stance phase during running (Ishikawa and Komi, 2007; Sousa et al., 2007). The present results agreed with these findings. Furthermore, an important finding of this study was that the extent of fascicle shortening during the eccentric phase significantly decreased after repeated SSC exercises (Table 1). This result was caused by a decline in active muscle stiffness after the fatigue task, since the muscle activities and stretch reflex during the drop jump did not change. Furthermore, it may be reasonable to consider that changes in fascicle behavior lead to an increase in the range of motion during the drop jump after repeated SSC exercises.

Another interesting result of the present study was that passive muscle stiffness significantly increased after repeated SSC exercises. According to previous findings (Jones et al., 1987; Howell et al., 1993), the passive muscle stiffness, evaluated by the relationship between the passive torque and joint angle during passive stretching, significantly increased following repeated eccentric exercises. Howell et al. (1993) gave two reasons for the exercise‐induced increase in the passive muscle stiffness: fluid accumulation within the muscles, and changes in the mechanical properties of connective tissues or cytoskeletal elements. In any case, the contribution of passive muscle stiffness to joint stiffness would be rather low, since passive torque was markedly lower than exerted torque during the drop jump.

In this study, we must draw attention to limitations and assumptions associated with the methodology followed. First, we used a previously reported moment arm length in order to calculate the Achilles tendon force. We cannot rule out the possibility that the moment arm length may change due to an acute increase in muscle thickness after the fatigue task. According to the finding of Sugisaki et al. (2015), the moment arm length increased by approximately 1.1 mm (5.5%) after 12 weeks of resistance training. In addition, they indicated that the effect of increases in the moment arm length may be negligible. Therefore, we considered that this point did not affect the main results of this study. Second, we considered that the contribution of MG during isometric plantar flexion did not change after the fatigue task. In the present study, no significant differences in the activation levels of LG and SOL during the measurements of active muscle stiffness and tendon properties were noted between before and after the fatigue task (data not shown). In addition, the coactivation level of TA was very small and did not change after the fatigue task (data not shown). Therefore, our muscle force calculation based on this assumption may have been valid for investigating changes in muscle and tendon properties after repeated SSC exercises. Third, we did not consider the effect of slack length during the measurement of active muscle stiffness. It is possible that the individual difference in slack length affects the measured variables. In the present study, however, we obtained the measured variables (torque and fascicle length) during submaximal contraction, but not relaxed condition. Furthermore, the torque values during relaxed condition (caused by inertia and passive elasticity) was subtracted from the measured torque during the measurement of active muscle stiffness. Considering these points, we considered that the influence of slack length on the measured variables was small. Fourth, the order of measurements would influence the measured variables. In this study, we performed the measurements in the order of joint stiffness, passive muscle stiffness, active muscle stiffness, and tendon stiffness. Ideally, we should perform the same experiments several times in order to obtain all the variables immediately after the fatigue task. However, we considered that this procedure was not realistic.

In conclusion, a decline in joint stiffness after repeated SSC exercises would be caused by changes in active muscle stiffness, but not those in tendon properties or neuromuscular activities. In a future study, we must establish a new endurance training protocol for active muscle stiffness and joint stiffness in order to enhance performance in athletic events such as long‐distance races.

## Conflict of Interest

The authors declare that they have no conflict of interest.
